# Retraction Note: Predictive value of microRNA-133a-3p for early urinary incontinence after radical prostatectomy for prostate cancer and its correlation with rehabilitation effects

**DOI:** 10.1186/s41065-025-00595-1

**Published:** 2025-10-17

**Authors:** Fan Yang, Qiuxia Qin, Juan Liu

**Affiliations:** Nursing Department, Tongji Hospital, Tongji Medical College, Huazhong University of Science and Technology, No. 1095 Jiefang Avenue, Wuhan, Hubei 430030 China

**Retraction Note: Hereditas 162, 75 (2025)**

**https://doi.org/10.1186/s41065-025-00443-2**

The Editor-in-Chief has retracted this article. After publication, concerns were raised about the authorship of this paper. Despite repeated requests, the Publisher was unable to obtain an explanation for these concerns, nor the source materials or ethics approval for the research reported in this article. The Editor-in-Chief, therefore, has lost confidence in the integrity of the article’s findings.

The authors agree with this retraction.

